# Optimal Design of Air Quality Monitoring Network and its Application in an Oil Refinery Plant: An Approach to Keep Health Status of Workers

**DOI:** 10.15171/hpp.2015.032

**Published:** 2016-01-30

**Authors:** Khaled ZoroufchiBenis, Esmaeil Fatehifar, Javad Ahmadi, Alireza Rouhi

**Affiliations:** Environmental Engineering Research Center, Faculty of Chemical Engineering, Sahand University of Technology, Tabriz, Iran

**Keywords:** Air Quality Monitoring Network, ISCST3 model, Multi-pollutant, Oil Refinery Plant

## Abstract

** Background: ** Industrial air pollution is a growing challenge to humane health, especially in developing countries, where there is no systematic monitoring of air pollution. Given the importance of the availability of valid information on population exposure to air pollutants, it is important to design an optimal Air Quality Monitoring Network (AQMN) for assessing population exposure to air pollution and predicting the magnitude of the health risks to the population.

** Methods: ** A multi-pollutant method (implemented as a MATLAB program) was explored for configur­ing an AQMN to detect the highest level of pollution around an oil refinery plant. The method ranks potential monitoring sites (grids) according to their ability to represent the ambient concentration. The term of cluster of contiguous grids that exceed a threshold value was used to calculate the Station Dosage. Selection of the best configuration of AQMN was done based on the ratio of a sta­tion’s dosage to the total dosage in the network.

** Results: ** Six monitoring stations were needed to detect the pollutants concentrations around the study area for estimating the level and distribution of exposure in the population with total network efficiency of about 99%. An analysis of the design procedure showed that wind regimes have greatest effect on the location of monitoring stations.

** Conclusion: ** The optimal AQMN enables authorities to implement an effective program of air quality management for protecting human health.

## Introduction

 Nowadays, air pollution is one of the serious problems that human beings are facing. Air pollutants are potentially hazardous to human health, plants, animals, materials and surrounding environment including building, roads, etc. The use of fossil fuels in most industries, transport and energy production can be considered as the major sources of atmospheric pollution. Emission of wide variety of pollutants such as sulfur dioxide and carbon monoxide in industrial plants such as oil refineries has made them as one of the largest sources of air pollution. The major health concerns associated with exposure to high concentrations of SO_2_ includes a higher incidence of respiratory diseases such as coughs, asthma, bronchitis and emphysema. Health effects of CO are generally considered in relation to carboxyhemoglobin levels in blood.^1-3^

 Measurement of air pollution and estimating its consequences is a way to manage and control of air pollution sources. It is critical to ensure the health of the residents and environment in the area surrounding an industrial plant. Establishing a proper Air Quality Monitoring Network (AQMN), therefore, plays an important role in developing policies and strategies for achieving air quality standards.^4-6^

 The design and operation of AQMN is determined by the objectives of the monitoring activities. The basic goal of air quality monitoring is the protection of human health and welfare. This broad goal has produced broad objectives, among which are the typical objectives reported by WHO, and are considered for industrial plants.^7^ They are determining compliance with national or international standards, developing policies and setting priorities for management actions, and developing and validating management tools such as models and geographical information systems.^8,9^ Generally the objectives of AQMN can be summarized in the terms of spatial representatively (i.e. siting criteria, including fixed or mobile sites and numbers of sites), time resolution, and measurement accuracy.^10^

 Designing optimal AQMN around industrial plants by finding the optimal number and location of monitoring stations will help environmental authorities and policy makers to manage and control air pollution sources. The optimal network provides the best representative cover and more air quality information while using minimum measurement devices, which reduces costs of implementation and maintenance of the network.

 Early works on finding number and locations of monitoring stations were based on the empirical judgments. Systematic approaches and statistical models for designing optimal AQMN were developed in recent years. Wang et. al.^11^ used the genetic algorithm to optimal design of AQMN. Al-Adwani et. al.^12^ proposed a surrogate-based optimization methodology for configuring the AQMN in an industrial area. They used multiple cell approach to create monthly spatial distribu-tions for the concentration of the pollutants. Chen et. al.^13^ used a multi-objective mixed-integer linear programming model to optimal design of an AQMN around a petrochemical plant. Elkamel et. al.^14^ used the multiple cell approach (MCA) for calculation of pollutant concentration in a study area and optimized the AQMN to achieve maximum spatial coverage and detection of violations over ambient standards. Kao and Hsieh4 used the concept of potential area to optimize AQMN for the Toufen Industrial District in Taiwan. Shahraiyni et. al.^15^ used the Artificial Neural Network (ANN) as the simulation tool in the optimization of location and number of air quality monitoring stations in Berlin. Mofarrah and Husain^16^ in joint work with Mofarrah et. al.^17^ optimized Riyadh City (Saudi Arabia) AQMN. Littidej et. al.^18^ applied mathematical model and GIS to determine a proper zone of air quality monitoring stations to monitor CO and NOx concentrations in a municipality area.

 Most of the techniques available in literature for designing AQMN are quite complex in nature and are suited to particular conditions and are not reusable for other cases.

 In this paper, a holistic and heuristic optimization method to represent multi-pollutant AQMN design is presented. Considering the high cost of stations (e.g., installation and maintenance), simultaneous measurement of several pollutants at one station is preferred. Besides the economic advantages of multi-pollutant AQMN design, estimating missing values will be practical using cross-correlations between the other pollutants. The proposed method was implemented as a MATLAB program, combined with Industrial Source Complex Short-Term 3 (ISCST3) model. The method is flexible and expandable which can consider multi-pollutant in the designing procedure and easily be used for various industrial areas. Using this method, the stations are being located in areas that have the maximum air pollutant concentration and high fluctuations of pollutant concentration, which leads to sensitivity of stations to pollutants sources. Therefore, the designed network provides better and more air quality information and detects highest levels of pollution while using minimum measurement devices.

 The proposed method was applied in Tabriz Oil Refining Company Plant, Tabriz, Iran as one of the proven sources of CO and SO_2_.

## Materials and Methods

 A quantitative procedure for the selection of air quality monitoring sites which utilizes dispersion model, historical meteorological data and probability calculations has been presented. The new terms such as contiguous potential monitoring area, cluster, station dosage, are a dosage and station efficiency were defined and used in the design procedure. The presented design procedure comprises analyses of the pollutants concentration over a set time period at a number of grid receptors. The ISCST3 model was used to identify the pollutants concentration in the study area and grid receptors. A threshold concentration which should not be exceeded was used to identify clusters. Each cluster is assigned a pollution dosage that is representative of that cluster. Station efficiency was calculated for each grid based on the station and area dosage. Finally the best location of monitoring stations were selected, one after the other, as the ones with the highest station efficiency.

###  Distribution of Pollutants Concentration

 The ISCST3 model was applied to simulate the dispersion of emitted pollutants from refinery stacks. The model has been specifically developed to simulate air pollution due to an industrial plant taking into account the effect of high stacks on the behavior of the pollutant plume.^19^ The Industrial Source Complex (ISC) model, as well as its short-term model (ISCST3), are among the most widely used and accepted models. This model includes a set of Gaussian plume-based models used for estimating ambient concentrations from point, area, and line sources up to a distance of 50 kilometers.^20,21^ Over the past 15–20 years, the ISCST3 model has been the preferred model for most regulatory modeling applications around the world.^22^ United States Environmental Protection Agency (US EPA) established the ISCST3 software for air pollution dispersion modeling, widely used in AQMN designing.^23-25^

 The ISCST3 model requires input information on emission sources and meteorological data. The emission sources information that needs to be input into the model are: stack characteristics such as height, internal diameter, exit gas velocity and temperature, pollutants (such as CO and SO_2_) emission rates and coordinates of sources.^19,26,27^ The ISCST model requires meteorological data to be used on an hourly basis format. The assumptions made in the ISCST3 model, are:^14,20,28,29^

 1.Steady state conditions;

 2.There is no reaction in the system;

 3.The emission inventories do not change by time at the specific days of mentioned scenarios;

 4.There is no deposition in the system;

 5.The effects of structures and buildings around pollution sources are neglected.

###  Formulation of the optimization method

 Initially, the study area (12000 × 10000m) was divided into grids of 200×200m and then ISCST3 model was employed to simulate distribution of pollutants concentrations in the study area. The results of ISCST3 model were used as the input of the optimization algorithm.

 The optimization algorithm can be broken down into the following steps:

 1. Allocation of all the results of ISCST3model to corresponding grids for each scenario^4,9,30^ and then normalization of concentrations for both CO and SO_2_ by scaling between 0 and 1.

 2. Aggregating of pollutant concentrations considering the suitable weighting factors.

 3. Identification of clusters based on the predefined threshold (TR). A cluster is the set of contiguous potential monitoring sites for which the overall normalized value exceeds the predefined threshold.

 4. Calculating Area Dosage; for any cluster, an area dosage is defined as the sum of the overall normalized values of each potential monitoring sites contained in that particular cluster.

 5. Calculating Station Dosage and Station Efficiency. Each potential monitoring site may be involved in more than one cluster; station dosage for *i*^th^ potential monitoring site is defined as the sum of the area dosages for all clusters containing the site *i*. The ratio of station dosage to sum of all the observed area dosages is defined as the station efficiency.

 6. Site selection

 The site selection process can be outlined as follows:

 6-1. Selecting the site with the highest station efficiency as the first monitoring station;

 6-2. Eliminating all clusters associated with the first monitoring station;

 6-3. Computing new station dosages and station efficiencies for the remaining sites;

 6-4. Selecting the most efficient site as the next monitoring station based on the new station dosages and station efficiencies;

 6-5. Continuing this process until the number of stations is adequate. There are two criteria for ending the process: 1) Budgetary constraint: in this case the number of monitoring stations is already known based on available budget, so the algorithm will find the best location of identified number of stations.

 2) Achieving maximum (or desired) total network efficiency: in this case there isn't any constraint for number of monitoring stations, so the algorithm will continue the process until to reach the maximum (or desired) amount of total network efficiency.

 Therefore, there are two modes in this section: (a) Specifying the number of stations at the start of the algorithm that can be based on budgetary constraints or policies of the environmental managers of industrial plant, (b) Running the program without any constraint for number of stations to reach network efficiency of 100%.^12,14,24^

## Results

###  Modeling of pollutants dispersion

 The hourly sequential meteorological data for wind speed, wind direction, ambient temperature, height of the mixing layer, and stability class, registered at a closest weather station of Tabriz Meteorological Organization was used as the input data to ISCST3 model. Table 1 shows the monthly average of meteorological data. The refinery includes 20 (CO and SO_2_) sources that disperse the exhaust pollutants over the surrounding area.

 Forty-two sets of data collected on 42 different days (information on emission sources & meteorological data) during the five years (from 2007 to 2012) were used in ISCST3 model to create ground level concentration values of CO and SO_2_ around the refinery plant for each scenario. The 42 data sets were selected in a way to achieve proper representation of the whole year. Considering the available data between the years 2008 to 2011, 34 data sets were selected from these available data. In order to have proper distributed data during the year (approximately with 9 or 10 days interval), the lack of data was filled by doing measurement in the year 2012 (8 data sets).

**Table 1:** Monthly average of meteorological data

	**Temperature** **(** ^o^ **C)**	**Prevailing wind** **speed (m/s)**			**Prevailing wind** **direction(degree)**		**Mixing height** **(m)**	
	**Value**	**SD**	**Value**	**SD**	**Value**	**SD**	**Value**	**SD**
January	-1.66	5.03	5.6	1.64	45	106	686	409
February	2.90	2.29	5.8	6.83	45	107	747	242
March	8.14	2.94	8.3	4.78	90	109	800	359
April	12.46	3.02	5.8	5.88	45	113	1034	430
May	18.00	2.76	8.3	4.57	270	96	1284	713
June	23.92	2.7	9.6	5.15	90	103	1530	917
July	26.86	2.59	11.0	5.12	90	77	1473	998
August	27.02	2.73	10.2	4.53	90	76	987	525
September	22.44	2.25	9.5	5.45	90	106	819	532
October	15.96	2.06	6.6	4.54	45	109	1094	613
November	7.40	1.94	5.1	3.84	45	117	785	413
December	1.98	1.69	5.4	7.48	45	115	863	373

**Fig. 1:**
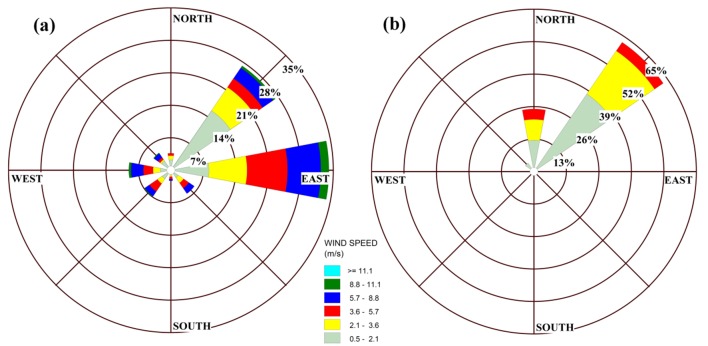
Windrose for the study area: (a) for the simulation days (b) for the desired day on March 2012

 Analysis of wind field data of last ten years (2002 to 2012) showed that dominant wind directions throughout the study area are Eastern (90 degree) and North Eastern (45 degree) and western (270 degree) winds. Besides, the windrose of collected data sets confirms the mentioned wind directions (Figure 1a).Figure 1b shows the windrose for a day on March 2012 (included in the data sets) which is selected as an example for investigation of pollutant dispersion around refinery.

Table 2 shows the coordinates, heights, and internal diameter of stacks as well as emission rate of CO and SO_2_, exit gas velocity and temperature which were measured by a flue gas analyzer (MRU Varioplus, Germany) on the same day.

**Table 2:** Stack characteristics and emission rates of CO and SO_2_ from refinery stacks

**Source**	**x** **Coord** ^*^	**y** **Coord**	**Stack height (m)**	**Stack diameter (m)**	**Stack** **temp(** ^o^ **C)**	**Gas velocity (m/s)**	**SO**_2_ **emission** **rate (gr/s)**	**CO** **emission** **rate (gr/s)**
1	0	655	73	3.5	194	6.5	44.80	2.13
2	24	655	73	3.5	198	5.45	152.88	0.49
3	46	655	73	3.5	194	6.35	27.98	0.73
4	125	674	36.6	1.9	635	4.2	2.23	0.04
5	128	854	43	3.57	513	6	0.80	0.59
6	139	854	36.6	2.18	552	14	4.22	0.12
7	162	677	36.6	0.92	490	7	0.40	0.31
8	162	685	46	1.81	355	3.9	0.07	0.04
9	162	694	46	2.18	261	4	0.57	1.92
10	220	667	36.6	2.2	525	7.22	14.15	14.85
11	246	667	36.6	4.35	618	12.18	45.11	2.68
12	272	667	36.6	2.35	374	1.1	0.15	12.99
13	365	676	52	2.52	426	3.9	5.11	0.26
14	406	672	73.2	3.58	248	4	7.00	0.47
15	396	672	36.6	1.5	262	8	6.74	0.35
16	429	677	52	2.38	207	8.2	10.63	0.53
17	435	677	53	1.5	244	6.5	4.24	0.64
18	436	817	36.6	1.58	215	6.2	2.82	0.28
19	351	667	36.6	3	401	6.55	3.05	0.09
20	200	515	60.8	2.35	316	6.6	46.17	20.22

* Coordinate system transformed to the center of simulation domain.

**Fig. 2:**
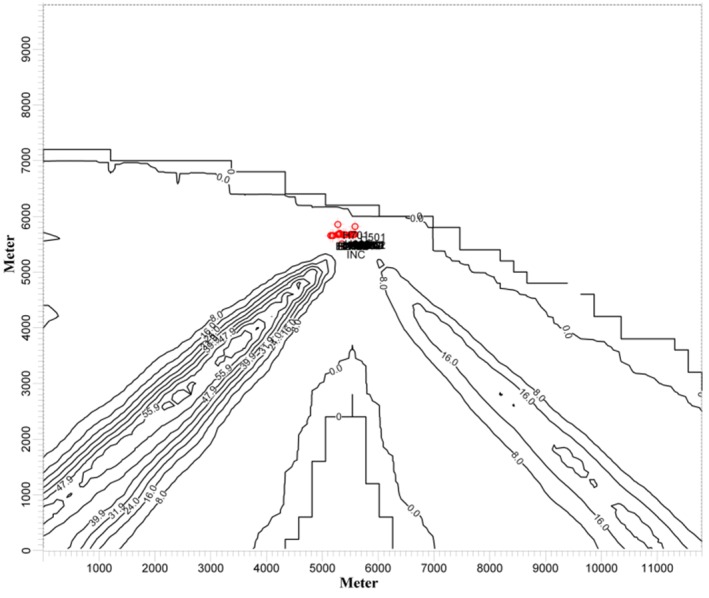
Results of a one-day simulation of SO_2_ in μg/m^3^

**Fig. 3:**
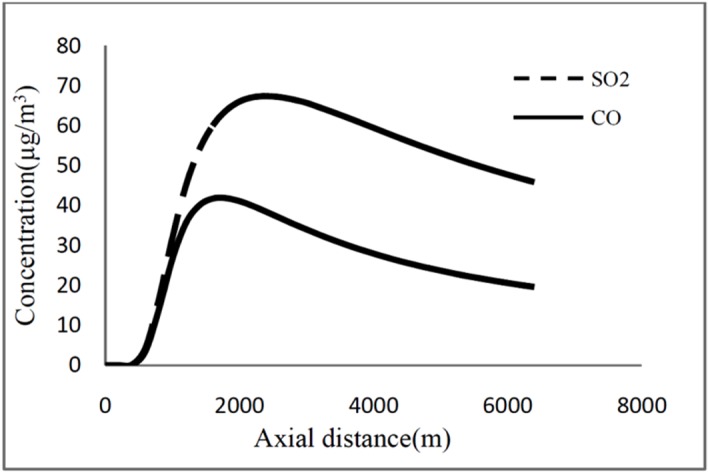
CO and SO_2_ concentrations estimated by model as function of axial distance at ground level

Figure 2 shows the results of a one-day simulation of SO_2_ in μg/m^3^.Figure 3 represents CO and SO_2_concentrations as a function of axial distance at ground level in the centerline of main plumes on the desired day.

###  Single pollutant monitoring network design

 The algorithm was implemented for different threshold values (*TR*). Given the air pollution dispersion, “potential zone” is recommended for locating the stations.

**Fig. 4:**
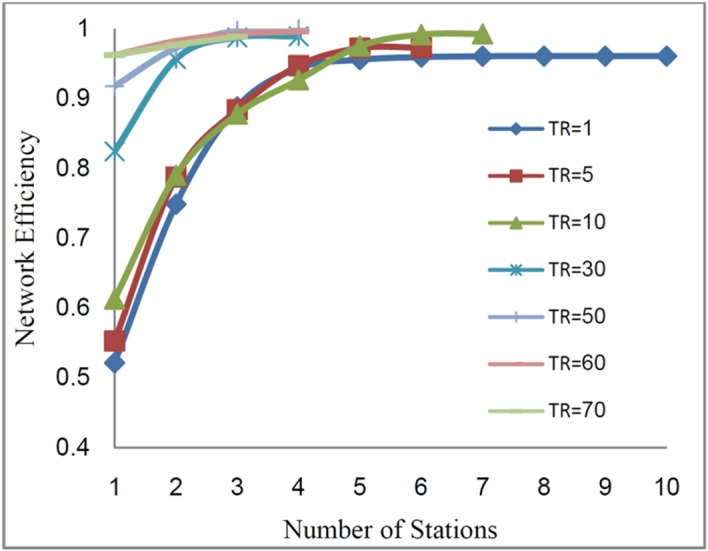
Network efficiency vs. number of stations for CO as a function of threshold values

 Potential zone is an area in which the pollutant concentrations are higher than a predefined threshold value. Figure 4 shows the network efficiency for different threshold values of CO.

 A network efficiency has been defined as a ratio of number of covered locations divided by the number of candidate locations, in which pollutant was appeared on them (higher than *TR* value) at least in one of simulations results of different conditions.

**Fig.5:**
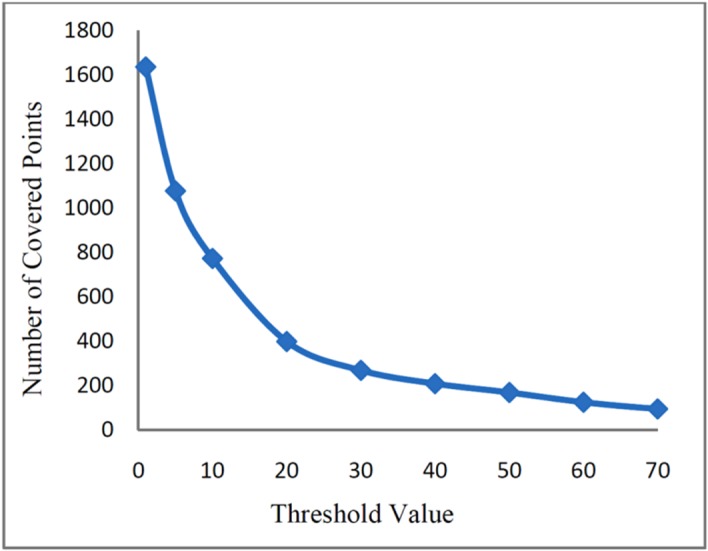
Network coverage as a function of threshold value (CO)

Figure 5 shows the number of covered points for different *TR* based networks (1<TR<70 µg/m^3^).

Table 3 shows the configuration of optimal network for *TR* value of 10 µg/m^3^. The number of stations location is based on sequential division of study area from 1 to 3000.

**Table 3:** Results of single-pollutant optimization for CO (*TR*=10)

**Station NO.**	**1**	**2**	**3**	**4**	**5**	**6**
Station location(coordinate)	45	421	1722	1716	912	1575
Number of covered points	272	111	114	99	127	49
Station efficiency	0.61	0.18	0.09	0.05	0.05	0.02
Network efficiency	0.61	0.79	0.88	0.93	0.98	0.99

 The value of 0.99 of network efficiency points out that the network covers 99 percent of points which have concentration value of more than TR = 10.

 In the case of SO_2_ for threshold values lower than 5, the network efficiency is lower than 1 and the required monitoring stations are fewer than 6. For the threshold values higher than 5, there is no slight change in the network efficiency but the network coverage decreases.

###  Multi-pollutant monitoring network design 

**Fig. 6:**
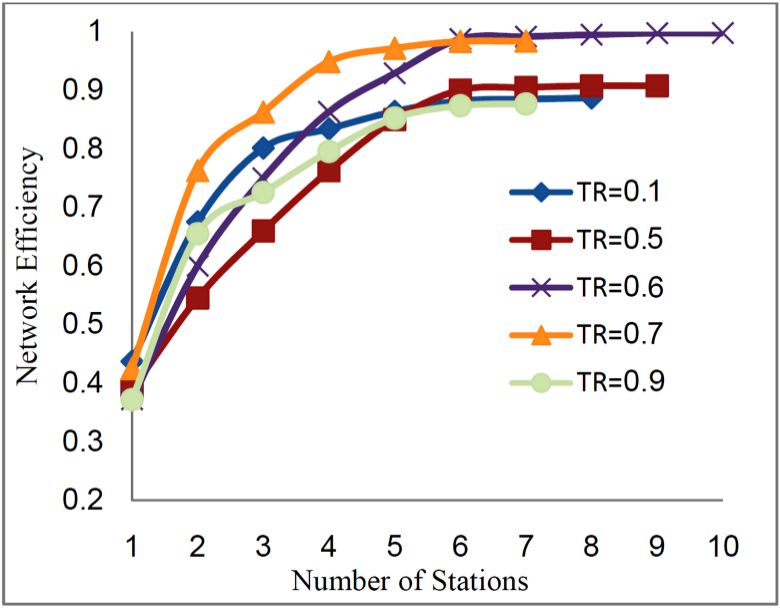
Network efficiency vs. number of stations for multi pollutant as a function of threshold values

 In this section, the threshold values vary from 0.1 to 1 (due to the normalization of concentration). Figure 6 shows the network efficiency for different values of *TR*.

**Fig. 7:**
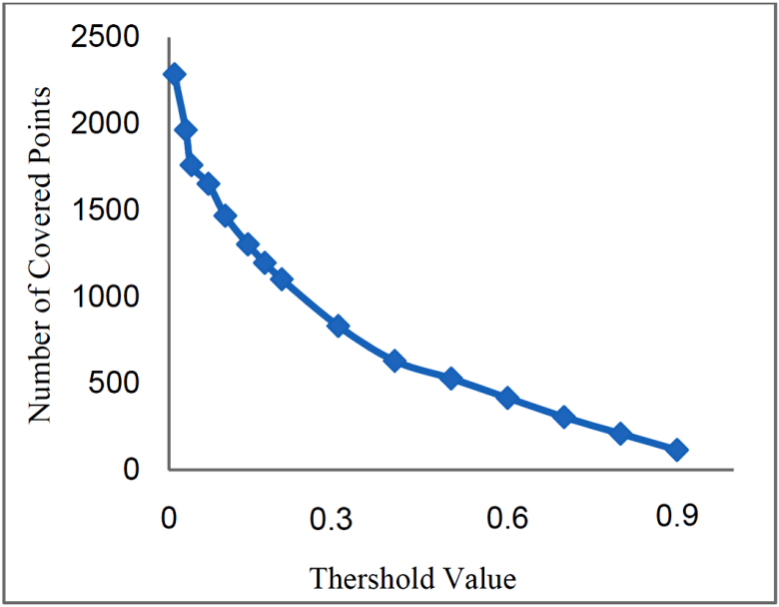
Network coverage and network efficiency as a function of threshold value (multi pollutant)

 For the threshold values higher than 0.6 the corresponding network efficiencies are close to 1. At the threshold value of 0.6 the network efficiency reaches the maximum of 0.99 and, as shown in Figure7, for higher *TR* values the network efficiency and network coverage begins to decrease.

**Fig. 8:**
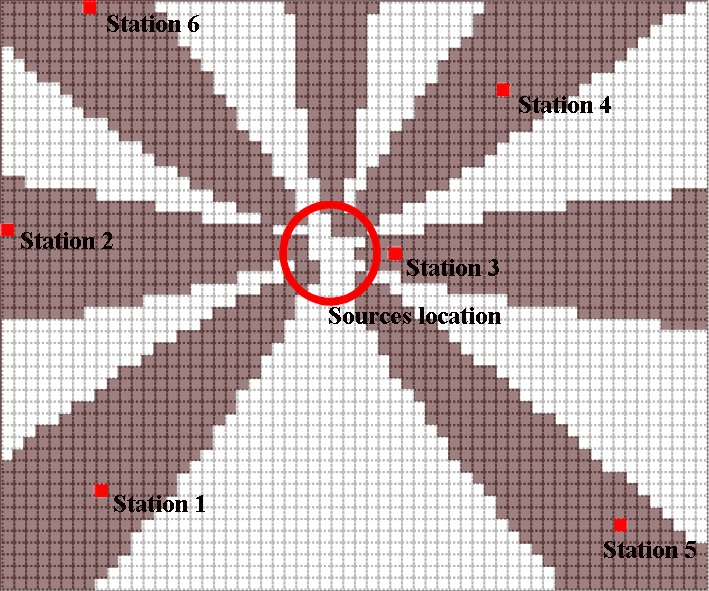
Location and coverage region of monitoring stations

Table 4 shows the results of multi-pollutant optimization for threshold value 0.6. Figure8 shows the location and coverage region of monitoring stations.

**Table 4:** Results of multi-pollutant optimization (*TR*=0.6)

**Station NO.**	**1**	**2**	**3**	**4**	**5**	**6**
Station location	442	20	1672	2108	2645	351
Number of covered points	302	193	226	300	268	215
Station efficiency	0.37	0.23	0.15	0.11	0.07	0.06
Network efficiency	0.37	0.60	0.75	0.86	0.93	0.99

## Discussion

###  Pollutants Distribution

 The dispersion of CO and SO_2_ emitted by the Tabriz Oil Refining Company was modeled using the ISCST3 air pollution model. The results of one day simulation (among 42 scenarios), which had almost constant wind direction and one main plume, were investigated to show the pattern of pollutant concentration and clarify the definition of potential zone. Regarding the prevailing wind direction of selected day (Figure 1b), the main plume was dispersed towards the southwest (Figure 2). Kao and Hsieh^4^, suggested that a monitoring station should be located in a potential zone, in which the pollutant concentration is larger than 90% of the maximum value. The clusters of each scenario is defined according to the potential zone, a set of land grids with pollutant levels exceeding the threshold value.^4,9,16^ According to the Figure 3, potential zone can be diagnosed from ground-level pollutant concentration profile. As an example for the SO_2_ in which the maximum level of concentration is67 µg/m^3^, the potential zone is downwind distance between1600 to 3800 m. In the case of CO with the maximum level of concentration of 41µg/m^3^the potential zone is downwind distance between 1200 to 2600 m (Figure 3). The length and the distance of potential zone from emission sources are dependent to pattern of pollutant concentration which is a function of meteorological conditions (includes temperature, wind speed and direction, mixing height, and data related to Pasquill’s stability classification) and emission rates.,^32^

###  Network Configuration

 In order to demonstrate the proposed methodology, it was used in two modes: designing the network based on 1) single pollutant monitoring network and 2) multi-pollutant monitoring network.

 In the case of single pollutant monitoring network design and in order to study the effect of *TR* on the network efficiency, the value of TR varied from 1 to 70*µg/m*^3^ (in the case of CO). The range of threshold value is selected based on the pollutant concentration level that occurred at different scenarios, and it can be determined from the reference level, considered for the AQMN. The selection of the threshold values (*TR*) varies depending on the case-by-case situation, like budget constraints, regional environmental regulations, meteorological condition and the purpose of the monitoring network.^4,7,14^ The results show that as the threshold value increased from 1 to 10, the network efficiency increased and reached the value of 0.99. For the higher value of threshold, the required monitoring stations decrease with increasing threshold value. The decreasing number of required monitoring stations is due to the shrinking of potential zone. In the other words, as the *TR* value decreases, the number of points which have the concentration level higher than *TR* value, and subsequently the number of required monitoring stations to cover these points decrease (Figure 5).^14,16^ Therefore, the optimal number and locations must be chosen with regards to high network coverage and network efficiency. The number of covered points for each station is the number of points included in potential zone over which the air quality data for a given monitoring location can be considered representative.^9,14,16^

 Considering that the network efficiency remains constant with increasing number of stations from 6 to 10, the6 monitoring stations corresponding to the threshold value of 10 are acceptable.

 In the case of multi-pollutants monitoring network design, for different *TR* values, as the number of stations increased from 1 to 6, the network efficiency also increased but for the number of stations more than 6 stations, the network efficiency remained constant. Thus, the 6 monitoring stations corresponding to the threshold value of 0.6 with the maximum network efficiency (99%) can be the best choice. In conclusion, establishing a network with more than 6 stations would not be economically justified for the study area.

 As shown in Figure 1a the prevailing wind directions throughout the study area were eastern, northeastern, western, and southwestern with the percentage of occurrence 41, 36, 7, and 5, respectively. The first four monitoring stations were spread mainly leeward of these prevailing wind directions(Figure 8) and also, as indicated at Table 3these stations provide network efficiency of 86% which confirms that wind regimes have the greatest effect on the location of monitoring stations.^4^ In other words, first four monitoring stations providing network efficiency of 37%, 23%, 15%, and, 11% respectively, were located at points which belong to more number of potential zones at different scenarios.^12,13,24^

###  Health Consequence of Optimal Network Design

 From the monitoring side, air pollution measurement is uneven and incomplete in many parts of the world, particularly in the industrial areas where impacts are the greatest.^5^Therefore, existing measurements do not fully address the evaluation of population exposure to air pollutants and the assessment of the resulting health effects.

 The air quality within Tabriz Oil Refining Company is measured seasonally at four non-optimal locations. These measurements may be descriptive of concentrations, but ambient observations alone fall short of identifying health effects and exposures populations. The optimal air quality monitoring network which can be roughly divided into better data coverage (e.g., more sites, more measurements) and better data quality (e.g., improved quality control protocols, more permanent measurement stations)^5,33^ has been presented for Tabriz Oil Refining Company by the proposed optimization procedure. This AQMN will enable the authorities to make appropriate plans for development of health promotion and prevention actions. Installation of monitoring stations and consequently taking appropriate decisions and actions based on provided data from monitoring stations will reduce the health effects of considered combustion pollutants.

 The combustion pollutants elicit several effects on the respiratory system (lower airways) including acute and chronic changes in pulmonary function, increased incidence and prevalence of respiratory symptoms, sensitization of airways to allergens, and exacerbation of respiratory infections, such as rhinitis, sinusitis, pneumonia, alveolitis, and Legionnaires’ disease. Taking control action to reduce the emission of combustion pollutants results in the reduction of health effects attributable to high level of ambient concentration SO_2_ and CO.^33,34,35^

 Considering that the terms of human population density and exposure of population to pollution were not taken into account directly as the AQMN objective in the optimization procedure, it is obvious that presented locations are around the high average concentration spots. Meteorological parameters change markedly in different months, which imposes some uncertainty on the distribution of pollutants and subsequently the selected location of monitoring stations.^4^ Exposure assessment is never complete when only a certain pollutant is measured. Several pollutants are synergistic and therefore it is mandatory to measure different pollutants at a common site.^7,9^ Given the mentioned facts, two suggestions can be made in the future studies: adding additional objectives to optimization procedure and reduction of uncertainties.

 Considering the demographic, climatic and geographic characteristics of the study area, different objectives such as maximization of the population protection, covering the areas with cultural heritage and sensitive receptors (e.g., schools, hospitals) and gaining maximum information on human exposure to high pollution can be included in the optimization procedure. In the optimization procedure, using the different meteorological and emission scenarios, as much as possible, will lead to the reduction of related uncertainties.

## Conclusions

 The described method was applied to the optimization of the air quality-monitoring network in Tabriz Oil Refining Company plant, for monitoring carbon monoxide and sulphur dioxide. The generated monitoring network provides maximum information about the multi-pollutants (in this study: CO and SO_2_) emitted from refinery stacks. For the area under study, 6monitoring stations are required for total network efficiency of 99%. The comparison between the results of single and multi-pollutants monitoring network design indicated that the proposed network design method had good efficiency in designing of multi-pollutants air quality monitoring network. So that the network efficiency of both designed networks were equal with the same number of monitoring stations. This heuristic leads to low cost and more information and provides the possibility of estimating missing values using cross-correlations between the pollutants.

 The proposed method is a suitable and effective method to design a proper air quality-monitoring network around an oil refinery that can be used for other industrial process plants such as petrochemical complexes and power plants.

## Acknowledgment

 This project was funded by the Tabriz Oil Refining Company. The authors would like to thank the company Research and Development Department for their kindly cooperation and for providing the case study.

## Conflict of interest

 The authors declare that there is no conflict of interests.
